# Adolescent Sexual Behavior Patterns in a British Birth Cohort: A Latent Class Analysis

**DOI:** 10.1007/s10508-019-01578-w

**Published:** 2020-01-06

**Authors:** Yin Xu, Sam Norton, Qazi Rahman

**Affiliations:** Department of Psychology, Institute of Psychiatry, Psychology and Neuroscience, King’s College London, 5th Floor Bermondsey Wing, Guys Hospital Campus, London, SE1 9RT UK

**Keywords:** Sexual behavior, Sexual attraction, Sexual orientation, Latent class analysis, ALSPAC

## Abstract

This study examined adolescent sexual behaviors patterns, and the consistency between sexual behavior and sexual orientation, in a prospective birth cohort. We used data on 5150 young people from the Avon Longitudinal Study of Parents and Children. Sexual orientation was assessed using a 5-point scale of sexual attraction at 15.5 years. Fourteen sexual activities were assessed using the Adolescent Sexual Activities Index at 13.5 and 15.5 years. Latent class analysis suggested four subgroups of adolescent sexual behaviors at 13.5: a “high-intensity sexual behaviors exclusively with other-sex, no same-sex intimacy” group (3.87%); a “moderate-intensity sexual behaviors exclusively with other-sex, no same-sex intimacy” group (16.57%); a “low-intensity sexual behaviors exclusively with other-sex, no same-sex intimacy” group (34.21%); and a “no sexual behavior” group (45.35%). There were five subgroups at 15.5 where four of them (23.42%, 18.37%, 28.12%, and 24.52%, respectively) were interpreted the same as at 13.5 years and a new “high-intensity sexual behaviors, some same-sex intimacy” subgroup (5.57%). Latent transition analysis showed approximately half the adolescents moved toward greater engagement in higher intensity sexual activities with other-sex at 15.5. Boys and girls who were in groups without same-sex intimacy were predominantly attracted to the other-sex, whereas there were moderate consistencies between same-sex intimacy and same-sex attraction for boys and low consistency for girls. Findings suggest that it may be important to include low-intensity sexual behaviors when assigning adolescents to sexual orientation groupings (via sexual behaviors) in order to reduce selection biases and increase statistical power via the increase in sample size.

**Electronic supplementary material:**

The online version of this article (10.1007/s10508-019-01578-w) contains supplementary material, which is available to authorized users.

## Introduction

### Subgroups of Adolescent Sexual Behavior

Sexual orientation refers to the degree to which a person is attracted to same- or opposite-sex members (Bailey et al., 2016). Sexual orientation is often measured using self-reports of sexual attraction toward men and women, sexual activity with men and women and self-identified sexual identity labels, or physiological measures including genital response, viewing time and pupil dilation (Bailey et al., 2016). While the state-of-the-art approach to measuring sexual orientation is to focus on sexual attractions or sexual “feelings,” there has been a tendency in epidemiological, applied and clinical sexual orientation research to measure sexual behavior (sexual acts, experiences with same- and opposite-sex partners). In contrast, basic sexual orientation research focuses on sexual attractions, fantasies and related psychological components (e.g., measures of approach or aversion to preferred and non-preferred sexual targets; Bailey et al., 2016). Much of the research on sexual orientation has also been conducted on adults. At this developmental stage, sexual attractions and behaviors may be more aligned than at earlier stages of development, such as adolescence or emerging adulthood (Gates, 2011; Savin-Williams & Diamond, 2004; Savin-Williams & Ream, 2007). In the past few decades, researchers have begun to focus on the development of adolescent sexual orientation.

The relationship between the different components of sexual orientation is poorly studied. Much of this research has focused on measurement of sexual intercourse (Zimmer-Gembeck & Helfand, 2008), though adolescents may engage in various sexual activities including kissing, touching genitals, oral sex and sexual intercourse during their sexual development (Hansen, Paskett, & Carter, 1999). In order to make conservative estimates of sexual orientation group membership, many studies have defined such groupings (e.g., being homosexual) on the basis of having engaged in sexual intercourse with the same-sex partners (e.g., Eisenberg & Resnick, 2006). Adolescents who are sexually active in sexual activities, except for sexual intercourse, may be excluded in research assigning adolescent sexual orientation only via sexual intercourse. At the very least, this introduces measurement error impacting precision. However, this strategy may also introduce substantial selection biases, bias parameter estimates of interest (e.g., associations between sexual orientation and a health outcome, or between a proposed causal factor and sexual orientation) and limit generalizability. For example, one British longitudinal study has found that only 330 boys had reported engaging in sexual intercourse in their sample (total sample size for boys is 2169), whereas many more boys reported engaging in other sexual activities (e.g., 883 boys had “petted under clothes”) (Li, Kung, & Hines, 2017). Accordingly, around 80% of the sample would have been excluded or misclassified if sexual orientation was measured only via sexual intercourse. Other studies using longitudinal cohorts support the notion that misclassification of sexual orientation is high when studies use single measures of sexual orientation or its components, especially sexual behavior (Savin-Williams & Ream, 2007).

Research on adolescent sexual development has also found that most heterosexual adolescents follow a progressive sexual trajectory from kissing and petting over the clothes to petting under the clothes and actually having sexual intercourse (de Graaf, Vanwesenbeeck, Meijer, Woertman, & Meeus, 2009; Shtarkshall, Carmel, Jaffe-Hirschfield, & Woloski-Wruble, 2009; Smiler, Frankel, & Savin-Williams, 2011). Subgroups of adolescents may follow different progress paths from less intimate (e.g., kissing) to more intimate (e.g., sexual intercourse) sexual behavior. For example, a cross-sectional study in the Netherlands found that about three quarters of heterosexual adolescents progressed gradually from kissing to having sexual intercourse in a stepwise manner, while the rest progressed rapidly into more intimate sexual behaviors (de Graaf et al., 2009).

A longitudinal study revealed three sexual development trajectories: a non-active sexual trajectory which included adolescents who engaged in no or minor sexual behaviors, a gradually sexually active trajectory which included adolescents who followed a stepwise progression from kissing to sexual intercourse, and a fast sexually active trajectory which included adolescents who rapidly progressed to more intimate sexual behaviors over a short period of time (Dalenberg, Timmerman, & van Geert, 2018). In contrast, non-heterosexual boys appear less likely to follow the stepwise progression from less intimate to more intimate sexual behaviors (Fish & Pasley, 2015; Smiler et al., 2011). For example, a study has found that boys with male partners were more variable in the age of first sexual intercourse, more likely to have sexual intercourse before kissing, and more likely to only have intercourse without experiencing kissing than boys with female partners (Smiler et al., 2011).

Different sexual behavior patterns may also be associated with different health outcomes and risky health behaviors. For example, a study has found that both adolescents with early sexual debut and adolescents with non-heterosexual identity were more likely to report depressive symptoms and more alcohol use than adolescents with late sexual debut (Fish & Pasley, 2015). Vasilenko, Kugler, Butera, and Lanza (2015) reported that group membership in riskier classes of sexual behavior was predicted by substance use and depressive symptoms. Among males, rates of sexually transmitted infections (STIs) increased with membership in classes with more risky sexual behaviors whereas females’ STI rates were consistent among all sexually active classes. Thus, understanding adolescents’ sexual behavior patterns could help basic sexual science and applied researchers to identify adolescents who are more likely to be at risk of particular negative health outcomes or engage in risky behaviors. This information could then inform the development of interventions targeted toward these groups. However, the range of sexual activities included in prior research is limited. Low-intensity or pre-intercourse activities including cuddling, laying down together and oral sex are poorly studied (especially in conjunction with more intimate sexual behaviors). Thus, more studies in well-characterized cohorts measuring various sexual activities are needed to identify potential subgroups of adolescent sexual behaviors.

### Consistency Between Sexual Behavior and Sexual Attraction

The consistencies among sexual orientation components are modest, perhaps because self-reported sexual identity and sexual behavior components are far more environmentally malleable than sexual attractions, desires and physiological responses (Bailey et al., 2016; Savin-Williams, 2009). Actions of people who engaged in same-sex behavior may reflect various motivations other than sexual attraction or are restricted by the availability (and desirability) of potential sex partners (Bailey et al., 2016). For example, situational same-sex sexual behavior has often been observed in settings that provide reduced access to members of the opposite-sex (e.g., in prisons and sex-segregated schools; see Green et al., 2003; Richters et al., 2012). About one quarter of men reported having been coerced into have same-sex sexual behavior in prison (Green et al., 2003). Another study using a large national dataset (National Survey of Family Growth) reported that 64.7% homosexual or bisexual men had intercourse with opposite-sex partner (Chandra, Mosher, & Copen, 2011). Once again, these prior studies suffered from potential selection bias, limited generalizability and reductions in statistical power due to the exclusion of participants who engaged in other sexual activities except sexual intercourse. Thus, sexual behaviors or activities and sexual attractions or sexual identity labels do not always align (Gates, 2011). Hence, further quantitative or data-driven research may help clarify how patterns or clusters of sexual behavior (often used for ease and practicality in large, epidemiological or cohort studies) relate to other indicators of sexuality or the consistency between sexual attraction and sexual behavior.

### The Current Study

Here we investigate whether there are subgroups of adolescent sexual behaviors at 13.5 and 15.5 years old separately, changes in sexual behavior patterns from 13.5 to 15.5 and the consistency between these adolescent sexual behaviors and sexual orientation (based on sexual attractions) in a British prospective birth cohort, the Avon Longitudinal Study of Parents and Children (ALSPAC). To achieve this, we use latent class analysis, a data-driven statistical approach which quantifies underlying and unmeasured group membership patterns among participants using the entire range of categorical observed variables. Then three-step latent transition analysis was estimated. In ALSPAC, available data were based on the measurement of 14 sexual activities at 13.5 and 15.5 and measurement of sexual orientation at 15.5. Given the exploratory nature of the latent class analysis and latent transition analysis, we do not have specific hypotheses regarding the predicted number of subgroups and transitions. But we hypothesized that there would be moderate consistency between sexual behavior and sexual attraction in our sample.

## Method

### Participants

Participants were part of the ALSPAC. All pregnant women with an expected date of delivery between April 1, 1991, and December 31, 1992, in the Bristol area of the South West of UK were eligible and invited to attend the ALSPAC. The initial sample recruited 14,541 (71.81% of the eligible sample) pregnant women who delivered 14,062 live-born children and 13,988 were alive at 1 year. Additional recruitment attempting to bolster the original sample with eligible cases who had failed to join the study at the beginning resulted in 15,458 fetuses with data collected from the age of seven onwards. Of this total sample of 15,458 fetuses, 14,775 were live births and 14,701 were alive at 1 year of age. Fifty-nine percent of the cohort attended the “Teen Focus” and have been followed four times between the age of 12.5 years old and 17 years old.

The 1991 UK Census was used to compare the population of mothers with infants under 1 year of age who were resident in Avon with those in the whole of the UK, and those of the ALSPAC who completed the 8-month postnatal questionnaire (Fraser et al., 2012). Mothers attended the ALSPAC were demographically similar to those of the rest of Great Britain (Fraser et al., 2012). The National Pupil Database (NPD) was further used to compare demographic information and educational attainment between children in the ALSPAC sample and children from a national sample. There was some indication that children in the ALSPAC sample had higher educational attainment at 16 years of age and to be of white ethnicity (Boyd et al., 2013). It was suggested that differences between children in the ALSPAC sample and children in the NPD can be associated with regional disparities and demographic changes within the UK since the initiation of the study (Boyd et al., 2013). Thus, children in the ALSPAC sample were relatively representative of children from across the UK. For more details, see Boyd et al. (2013). The study Web site contains details of all the data, which are available through a fully searchable data dictionary: http://www.bris.ac.uk/alspac/researchers/data-access/data-dictionary/. Ethical approval for the study was obtained from the ALSPAC Law and Ethics Committee, and King’s College London Psychiatry, Nursing & Midwifery research ethics subcommittee.

The current study analyzed ALSPAC data reported by children at 13.5 and 15.5 years old. The response rates were around 55.00% and 52.00% at 13.5 and 15.5 years old, respectively. One study showed ASLAPC children who had higher maternal education were less likely to drop out (Howe, Galobardes, Tilling, & Lawlor, 2011). Adolescents who reported a valid response of sexual orientation and at least one item of Adolescent Sexual Activities Index at 13.5 or 15.5 years old were included here, which resulted in a sample of 4604 adolescents (2172 boys and 2432 girls) at 13.5 years old and 5150 adolescents at 15.5 years old (2406 boys and 2744 girls).

### Measures

#### Sexual Orientation

At age of 15.5 years old, adolescents were required to answer the question: “Please choose the description that best fits how you think about yourself” on a 5-point Kinsey-like scale: 1 = 100% heterosexual, 2 = mostly heterosexual but also attracted to the same-sex, 3 = bisexual (equally attracted to both-sexes), 4 = mostly homosexual but also attracted to the other-sex, 5 = 100% homosexual, 6 = not sexually attracted to either sex and 7 = not sure. This was done via computer to promote disclosure of sensitive personal information.

Such 5-point scales of sexual orientation (attractions) have been used in large studies of adolescents (Ott, Corliss, Wypij, Rosario, & Austin, 2011; Saewyc, Skay, Bearinger, Blum, & Resnick, 1998). Five-point item shows good stability in adolescents (Ott et al., 2011), associations with same-sex sexual behavior (Saewyc et al., 1998) and low nonresponse rates compared with other components of adolescent sexual orientation (Saewyc et al., 2004). Men and women recall first having feelings of sexual attraction at approximately age 10, on average (McClintock & Herdt, 1996). One study reported first awareness of same-sex attraction at approximately 15 years (Calzo, Antonucci, Mays, & Cochran, 2011), while other studies report even earlier recalled mean age of same-sex attractions (e.g., Floyd & Bakeman, 2006). Therefore, it appears to be appropriate to begin measuring sexual attractions at 15.5 years old. As mostly heterosexuals, bisexuals, mostly gay/lesbians are sometimes reported to display unique patterns of attraction and sexual behavior (Vrangalova & Savin-Williams, 2012), we treated them as separate groups. Latent class analysis may give us more quantitative information about the sexual behavior patterns of adolescents who chose “not sexually attracted to either sex” or “not sure,” they were also included in the analyses. As a result, 2157 100% heterosexual boys (89.64%), 146 mostly heterosexual boys (6.07%), 29 bisexual boys (1.21%), 14 mostly homosexual boys (0.58%), 16 homosexual boys (0.67%), 8 boys who were not sexually attracted to either sex (0.33%), 36 boys who were not sure about his sexual orientation (1.50%), 2310 100% heterosexual girls (84.18%), 295 mostly heterosexual girls (10.75%), 56 bisexual girls (2.04%), 13 mostly homosexual girls (0.47%), 4 homosexual girls (0.15%), 9 girls who were not sexually attracted to either sex (0.33%), 57 girls who were not sure about her sexual orientation (2.08%) were included.

#### Sexual Behavior

When adolescents were 13.5 and 15.5 years old, they were asked to report whether they had engaged in any of fourteen sexual activities from the Adolescent Sexual Activities Index (ASAI; Hansen et al., 1999). Those sexual activities were presented in order from low (e.g., hug or hold hand) to high (e.g., have oral sex or have sexual intercourse) intensity. Adolescents were required to report whether they had engaged in each sexual activity in the past year, and the sex of the person with whom they engaged in each activity. Adolescents who reported not having engaged in a particular sexual activity received a score of 0 on that activity, those who reported engaging in the activity with the other-sex received a score of 1 on that activity, those who reported engaging in the activity with both-sexes received a score of 2 on that activity, and those who reported engaging in the activity with the same-sex received a score of 3 on that activity. ASAI is a validated self-report measure and has high internal consistency (Cronbach’s *α* = 0.93) (Hansen et al., 1999).

### Statistical Analysis

#### Missing Data

Thirty-three (0.72%) out of 4604 adolescents at 13.5 and 35 (0.68%) out of 5150 adolescents at 15.5 were with incomplete information on the sexual behavior within the analysis sample. A total of 546 (10.60%) adolescents with sexual behavior information were available only at 15.5 years old. Missing data were handled via full information maximum likelihood estimation with robust standard errors (MLR) in Mplus 7.4. This approach makes the assumptions that data are missing at random conditional on variables related to missingness being included in the analysis.

#### Latent Class Analysis

Latent class analysis is used to identify the underlying subgroups in a population across a set of theoretically selected variables (McLachlan & Peel, 2004). Through latent class analysis, we can evaluate whether there are meaningful subgroups of adolescents who show the similar sexual behavior patterns. A cross-sectional latent class analysis was estimated for age 13.5 and 15.5 separately. Fourteen items of ASAI (0 = no, 1 = other-sex. 2 = both-sexes and 3 = same-sex) as the manifest indicators of latent class memberships were used in the latent class analyses. In order to identify the best-fitting model that explained our data and optimally described the heterogeneity, a series of latent class models with increasing numbers of classes were fit to the data in Mplus 7.4. We started with a single-class model and fitted successive models with an increasing number of latent classes (one at a time) until the results were no longer interpretable. The best-fitting model was determined by the interpretability of the model, sample size of each latent class and model fit statistics including Akaike Information Criterion (AIC), Bayesian Information Criterion (BIC), adjusted BIC (ABIC), Bootstrapped likelihood ratio test (BLR test), Lo-Mendell-Rubin likelihood ratio test (LMR LR test), and adjusted LMR LR test (ALMR LR test). Classification uncertainty was quantified by the calculation of entropy based on the posterior probabilities of latent class membership. Information criteria present likelihoods penalized for model complexity, where a model with smaller values of AIC, BIC, and ABIC fits better than a model with higher values (Asparouhov & Muthén, 2012). For the LMR LR test, ALMR LR test, and BLR test, a significant *p* value indicates that the *k* classes model fit the data better than the *k* − 1 classes model (Asparouhov & Muthén, 2012). A model with high value of classification entropy indicates greater classification certainty and thus better model fit. Analyses were computed in a stepwise fashion considering models with one-to-eight latent classes (as recommended by Asparouhov & Muthén, 2012). In order to ensure the model converged at its global maxima, models were estimated multiple times using 500 random sets of starting values to ensure the best log-likelihood values were replicated across the different models. Once the best-fitting model was determined, adolescents were assigned their most likely latent class membership representing their sexual behavior profile.

As a comparison, we conducted another latent class analysis with 5-point scale of sexual attraction at 15.5, a dichotomous variable regarding whether adolescents were not sure about their sexual orientation at 15.5, a dichotomous variable regarding whether adolescents were not sexually attracted to either sex at 15.5, and ASAI items as indicators of latent class membership at 15.5 (see Supplemental Text, Supplemental Table 1 and Supplemental Table 2 for details). Different number of classes found in the supplemental analysis is simply the natural consequence of adding additional predictors into the latent class analysis, but the results were essentially the same regarding the consistency between sexual behaviors and sexual attractions.

#### Latent Transition Analysis

Through latent transition analysis, we can quantify the changes of sexual behavior patterns in a matrix of transition probabilities between consecutive time points. Here we investigated the changes in sexual behavior from 13.5 to 15.5 years old. Latent class analyses at 13.5 and 15.5 years old showed that different numbers of latent classes were identified over time. Although there were four latent classes that are almost the same at both time points, the item-response probabilities were slightly different (e.g., class 2 between 13.5 and 15.5), which implies some measurement variance. Thus, a three-step latent transition analysis with measurement variance, which relaxed the measurement parameters of classes to be freely estimated, was estimated in Mplus 7.4 (Asparouhov & Muthén, 2014; Nylund-Gibson, Grimm, Quirk, & Furlong, 2014). In the first step, a latent class model was estimated for age 13.5 and 15.5 separately using only the fourteen items of sexual activities. In the second step, the most likely latent class memberships for age 13.3 and 15.5 were derived using the latent class posterior distributions obtained from the first step. In the final step, the most likely class memberships at 15.5 was regressed on the most likely class memberships at 13.5 with the misclassification in the second step being taken into account. This method was used to incorporate the measurement error from the determination of most likely latent class (Kamata, Kara, Patarapichayatham, & Lan, 2018). Since 546 (10.60%) adolescents with sexual behavior information and latent class memberships were available only at 15.5 years old, 4604 adolescents with latent class memberships available at both 13.5 and 15.5 years old were included in the latent transition analysis.

#### Regression Analysis

Analyses were performed in Stata 15.0. All analyses were carried out separately for boys and girls. A univariate probability-weighted multinomial logistic regression was estimated with latent class membership regressed on sex to test sex differences in the latent class membership. In order to test the consistency between sexual orientation (here, attraction) and sexual behavior, a univariate probability-weighted linear regression was estimated with sexual orientation (5-point scale without “not sure” and “no sexual attractions”) regressed on latent class membership. The maximum posterior probability of an observation was added into the regression as a sampling weight (Kamata et al., 2018). This method was used to incorporate the measurement error from the determination of most likely latent class (Kamata et al., 2018). We chose the linear regression over ordered logistic regression (sexual orientation was treated as an ordinal variable) or multinomial logistic regression (sexual orientation was treated as a nominal variable) to test the consistency between sexual behavior and sexual attractions given some small cell sizes of sexual orientation stratified by sex and latent class memberships (e.g., no boys from Class 3 at 15.5 years old identified themselves as bisexual or mostly homosexual).

While no formal power calculation was conducted, it is possible to consider power to detect a meaningful effect. The sample size exceeds the sample size requirement of detecting *R*^2^ of 0.09 at the 5% significance level and 80% power. Converted to the Cohen’s *d* metric, a *R*^2^ of 0.09 equates to a Cohen’s *d* of .63, which is generally considered to be a medium effect (Borenstein, Hedges, Higgins, & Rothstein, 2009).

## Results

### Latent Class Analysis of Sexual Behavior

**Fig. 1:**
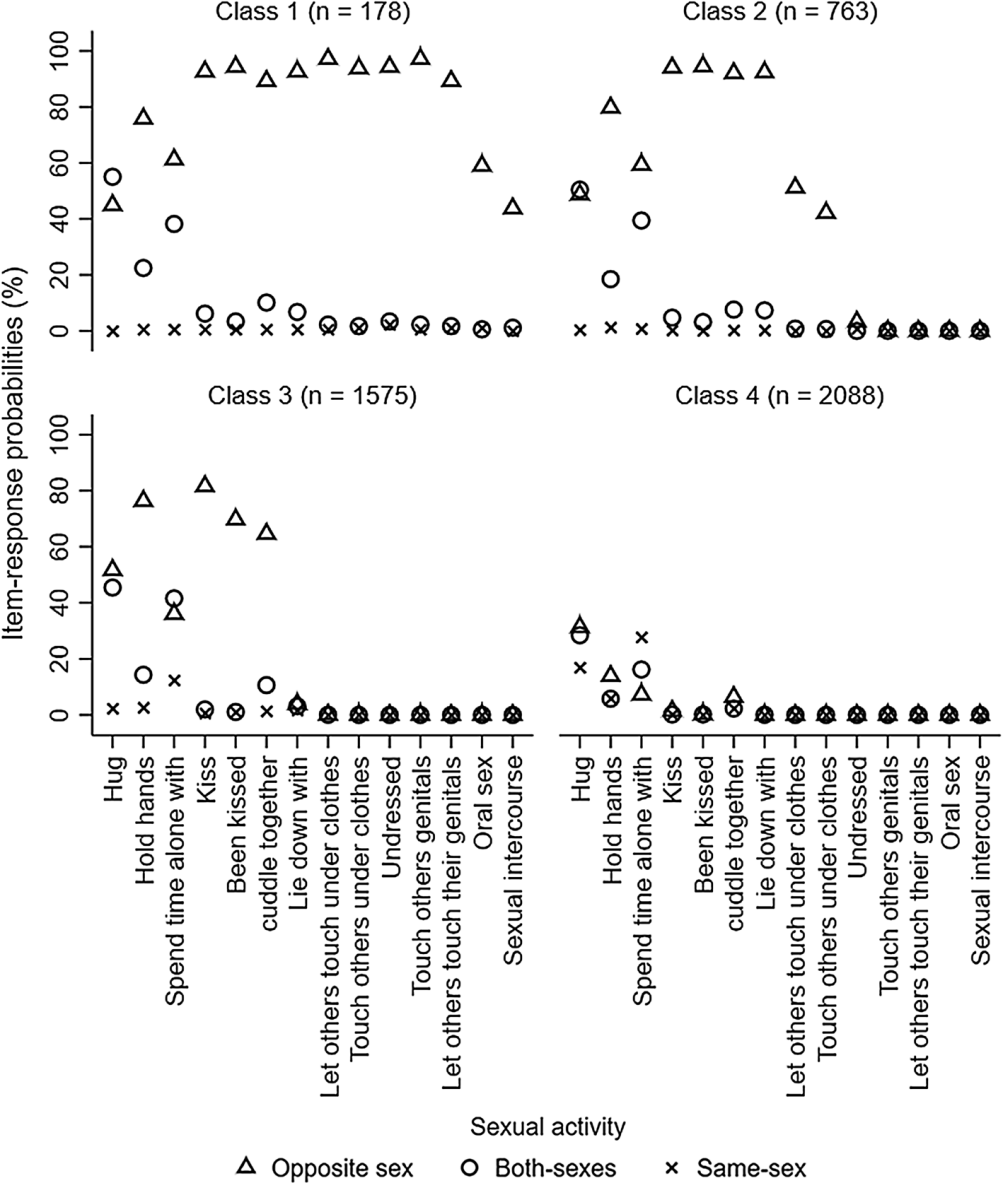
Item-response probabilities for four classes model of adolescent sexual behavior at 13.5 years. *Note* This figure shows the probabilities of adolescents engaging in sexual activity with the other-sex, both-sexes, same-sex partners at 13.5 years stratified by latent class memberships and sexual activities

We compared models with one through to eight latent classes (Table 1). For latent class models at 13.5 years old, the values of AIC, BIC, and ABIC decreased from the one class model to the eight classes model. The results of LMR LR and ALMR LR tests suggested that the four classes model was the most optimal. Based on a careful inspection of the four, and five classes models, we selected the four classes model. This model was chosen over the five classes model because the classes were more interpretable and all classes were qualitatively distinct from one another based on item-response probabilities (e.g., both Class 3 and 4 from the five classes model were marked by a high probability of kissing the other-sex partners only (see Fig. 1 and Supplemental Figure 1 for the comparison among four and five classes models).

**Table 1:** Model fit statistics for latent class analysis of adolescent sexual behavior with 1–8 classes stratified by age

Age	Number of classes	AIC	BIC	ABIC	LMR LR test	ALMR LR test	BLRT	Entropy
13.5	1	76,252.76	76,516.58	76,386.30				
	2	60,314.86	60,848.94	60,585.20	< .001	< .001	< .001	.94
	3	55,383.53	56,187.87	55,790.67	< .001	< .001	< .001	.93
	4	53,424.66	54,499.25	53,968.59	< .001	< .001	< .001	.92
	5	52,103.72	53,448.57	52,784.44	.587	.587	< .001	.91
	6	51,181.10	52,796.21	51,998.62	.793	.793	< .001	.87
	7	50,452.07	52,337.43	51,406.39	.764	.764	< .001	.86
	8	50,115.72	52,271.34	51,206.83	.760	.760	< .001	.86
15.5	1	117,157.39	117,432.35	117,298.89				
	2	86,981.50	87,537.97	87,267.87	< .001	< .001	< .001	.97
	3	75,692.19	76,530.18	76,123.44	< .001	< .001	< .001	.96
	4	72,435.07	73,554.57	73,011.19	.019	.019	< .001	.96
	5	69,543.05	70,944.06	70,264.04	.774	.774	< .001	.95
	6	67,708.95	69,391.47	68,574.80	.765	.765	< .001	.93
	7	66,623.37	68,587.39	67,634.09	.395	.396	< .001	.93
	8	65,866.07	68,111.60	67,021.66	.756	.756	< .001	.92

Using item-response probabilities (Table 2; Fig. 1), we interpreted the four classes as: “high-intensity sexual behaviors, no same-sex intimacy” (Class 1), “moderate-intensity sexual behaviors, no same-sex intimacy” (Class 2); “low-intensity sexual behaviors, no same-sex intimacy” (Class 3) and “no sexual behavior” (Class 4). The “high-intensity sexual behaviors, no same-sex intimacy” class contained 3.87% % (*n* = 178) of participants and was marked by a high probability of engaging in all sexual activities exclusively with other-sex partners. The “moderate-intensity sexual behaviors, no same-sex intimacy” class contained 16.57% (*n* = 763) of participants and was characterized by a high probability of kissing and touching with other-sex partners only. The “low-intensity sexual behaviors, no same-sex intimacy” class contained 34.21% (*n* = 1575) of participants and was characterized by a high probability of kissing other-sex partners only. The “no sexual behavior” class contained 46.35% (*n* = 2088) of participants who had a high probability of reporting having not engaged in any sexual activities.

**Table 2:** Item-response probabilities (%) based on the posterior probabilities for four classes model of adolescent sexual behavior at 13.5 years

Sexual behavior	Latent class membership			
	Class 1 (*n* = 178)	Class 2 (*n* = 763)	Class 3 (*n* = 1575)	Class 4 (*n* = 2088)
Hug				
No	.00	0.40	0.50	23.30
Other-sex	44.90	48.40	52.20	30.80
Both-sexes	55.10	50.70	45.10	28.50
Same-sex	0.00	0.50	2.20	17.50
Hold hands				
No	1.10	0.50	7.10	75.20
Other-sex	75.80	79.80	76.30	12.70
Both-sexes	22.50	18.50	13.90	6.00
Same-sex	0.60	1.20	2.70	6.10
Spend time alone with				
No	0.00	0.70	10.20	48.70
Other-sex	61.30	58.70	36.80	6.70
Both-sexes	38.20	39.40	41.20	16.10
Same-sex	0.60	1.20	11.80	28.50
Kiss				
No	0.60	0.90	18.20	97.70
Other-sex	92.60	94.10	79.30	1.90
Both-sexes	6.20	4.70	1.90	0.20
Same-sex	0.60	0.30	0.60	0.20
Been kissed				
No	1.70	3.10	29.20	99.50
Other-sex	94.30	93.60	68.70	0.30
Both-sexes	3.40	3.10	1.10	0.20
Same-sex	0.60	0.20	1.00	0.00
Cuddle together				
No	0.00	0.00	24.30	89.20
Other-sex	89.30	91.50	64.60	5.90
Both-sexes	10.10	8.20	9.80	2.50
Same-sex	0.60	0.30	1.30	2.40
Lie down with				
No	0.00	0.00	90.00	99.90
Other-sex	92.70	91.00	5.90	0.00
Both-sexes	6.70	8.60	2.50	0.10
Same-sex	0.60	0.40	1.60	0.00
Let others touch them under clothes				
No	0.00	46.10	99.70	100.00
Other-sex	97.20	53.10	0.20	0.00
Both-sexes	2.20	0.80	0.10	0.00
Same-sex	0.60	0.00	0.00	0.00
Touch others under clothes				
No	3.40	55.50	99.90	100.00
Other-sex	93.80	43.80	0.00	0.00
Both-sexes	1.70	0.70	0.00	0.00
Same-sex	1.10	0.00	0.10	0.00
Undressed				
No	0.00	95.80	100.00	100.00
Other-sex	94.40	3.40	0.00	0.00
Both-sexes	3.40	0.00	0.00	0.00
Same-sex	2.20	0.80	0.00	0.00
Touched others genitals				
No	0.00	100.00	100.00	100.00
Other-sex	97.20	0.00	0.00	0.00
Both-sexes	2.20	0.00	0.00	0.00
Same-sex	0.60	0.00	0.00	0.00
Let others touch their genitals				
No	7.90	100.00	100.00	100.00
Other-sex	89.30	0.00	0.00	0.00
Both-sexes	1.70	0.00	0.00	0.00
Same-sex	1.10	0.00	0.00	0.00
Oral sex				
No	39.30	100.00	100.00	100.00
Other-sex	59.00	0.00	0.00	0.00
Both-sexes	0.60	0.00	0.00	0.00
Same-sex	1.10	0.00	0.00	0.00
Sexual intercourse				
No	54.80	100.00	100.00	100.00
Other-sex	44.10	0.00	0.00	0.00
Both-sexes	1.10	0.00	0.00	0.00
Same-sex	0.00	0.00	0.00	0.00

No odds ratios were presented for comparing item-response probabilities across different classes given lots of zero cell sizes. The four classes were interpreted as: “high-intensity sexual behaviors, no same-sex intimacy” (Class 1), “moderate-intensity sexual behaviors, no same-sex intimacy” (Class 2); “low-intensity sexual behaviors, no same-sex intimacy” (Class 3); and “no sexual behavior” (Class 4)

**Fig. 2:**
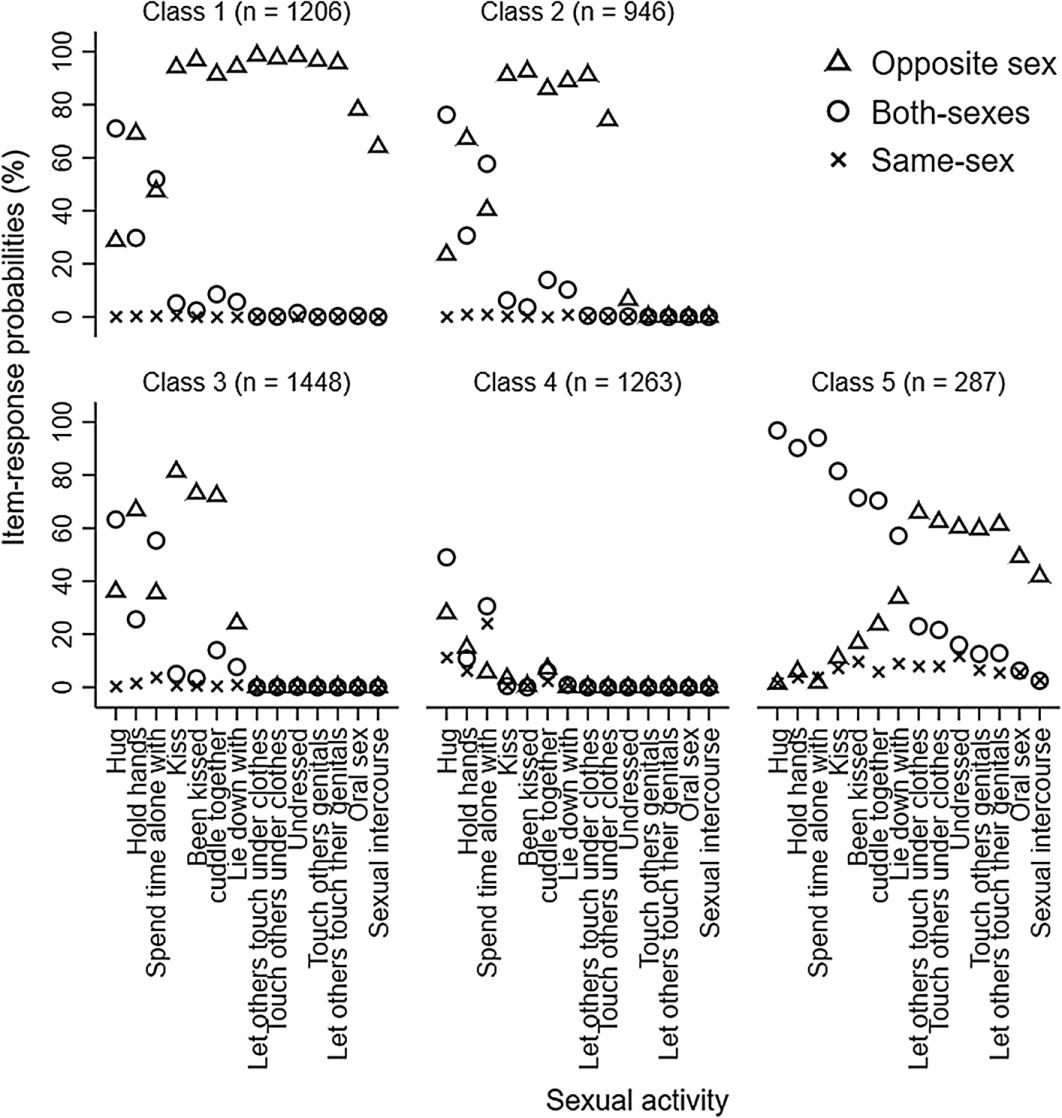
Item-response probabilities for five classes model of adolescent sexual behavior at 15.5 years. *Note* This figure shows the probabilities of adolescents engaging in sexual activity with the other-sex, both-sexes, same-sex partners at 15.5 years stratified by latent class memberships and sexual activities

For latent class models at 15.5, the values of AIC, BIC and ABIC also decreased from the one class model to the eight classes model. The results of LMR LR and ALMR LR tests suggested that the four classes models were the most optimal. Based on a careful inspection of the four, five, and six classes models, we selected the five classes model. This model was chosen over the four and six classes model because the classes were more interpretable and all classes were qualitatively distinct from one another based on item-response probabilities. For example, compared to the four classes model, the five classes model added a new class which was marked by a high probability of kissing and touching with other-sex partners only; compared to the five classes model, both Class 2 and 5 from the six classes model were marked by a high probability of kissing the other-sex partners only. (see Fig. 2, Supplemental Figure 2 and Supplemental Figure 3 for the comparison among four, five and sixes classes models).

Using item-response probabilities (Table 3; Fig. 2), we interpreted the five classes as: “high-intensity sexual behaviors, no same-sex intimacy” (Class 1, *n* = 1206, 23.42%), “moderate-intensity sexual behaviors, no same-sex intimacy” (Class 2, *n* = 946, 18.37%); “low-intensity sexual behaviors, no same-sex intimacy” (Class 3, *n* = 1448, 28.12%); “no sexual behavior” (Class 4, *n* = 1263, 24.52%); and “high-intensity sexual behaviors, some same-sex intimacy” (Class 5, *n* = 287, 5.57%). The Class 1 to Class 4 found at 15.5 years old were interpreted as the same as the Class 1 to Class 4 found at 13.5 years old. There was a new class found on 15.5 years old, the “high-intensity sexual behaviors, some same-sex intimacy” class which was marked by a high probability of engaging in high-intensity behaviors exclusively with the other-sex partners such as genital touching and oral sex, and low-intensity sexual activities with both-sexes partners.

**Table 3:** Item-response probabilities (%) based on the posterior probabilities for five classes model of adolescent sexual behavior at 15.5 years

Sexual behavior	Latent class membership				
	Class 1 (*n* = 1206)	Class 2 (*n* = 946)	Class 3 (*n* = 1448)	Class 4 (*n* = 1263)	Class 5 (*n* = 287)
Hug					
No	.10	.10	.20	11.20	.00
Other-sex	28.60	23.40	36.50	28.30	1.40
Both-sexes	71.10	76.40	62.80	49.20	96.90
Same-sex	.20	.10	.50	11.30	1.70
Hold hands					
No	.80	1.00	6.80	67.30	.00
Other-sex	68.90	67.20	65.90	16.00	6.40
Both-sexes	30.00	30.90	25.50	10.60	89.60
Same-sex	.30	.90	1.80	6.10	4.00
Spend time alone with					
No	.30	.80	5.80	38.90	.40
Other-sex	47.20	40.50	35.40	5.60	1.80
Both-sexes	52.10	57.80	54.60	31.50	94.00
Same-sex	.40	.90	4.20	24.00	3.80
Kiss					
No	.20	1.60	13.40	95.40	.00
Other-sex	93.60	91.90	80.80	3.40	12.50
Both-sexes	5.70	6.10	5.00	.50	80.20
Same-sex	.50	.40	.80	.70	7.30
Been kissed					
No	.70	3.20	23.80	98.60	2.20
Other-sex	96.30	93.10	72.10	0.60	18.00
Both-sexes	2.90	3.50	3.40	.00	70.00
Same-sex	.10	.20	.70	.80	9.80
Cuddle together					
No	.00	.00	14.40	83.80	.00
Other-sex	91.50	86.00	70.30	8.70	23.00
Both-sexes	8.50	14.00	14.80	5.10	71.10
Same-sex	.00	.00	.50	2.40	5.90
Lie down with					
No	.00	.00	69.90	98.20	.00
Other-sex	94.60	89.00	21.60	.10	32.20
Both-sexes	5.40	10.20	7.50	.90	58.60
Same-sex	.00	.80	1.00	.80	9.20
Let others touch them under clothes					
No	1.30	13.80	99.70	99.80	3.00
Other-sex	98.60	85.70	.30	.00	65.90
Both-sexes	.10	.40	.00	.00	23.10
Same-sex	.00	.10	.00	.20	8.00
Touch others under clothes					
No	2.40	29.70	99.90	99.90	8.00
Other-sex	97.50	69.80	.00	.00	62.50
Both-sexes	.10	.40	.10	.00	21.40
Same-sex	.00	.10	.00	.10	8.10
Undressed					
No	.00	93.30	99.90	99.90	12.60
Other-sex	98.30	6.30	.00	.00	59.20
Both-sexes	1.50	.20	.01	.00	16.20
Same-sex	.20	.20	.00	.10	12.00
Touched others genitals					
No	3.20	100.00	100.00	100.00	21.90
Other-sex	96.70	.00	.00	.00	58.80
Both-sexes	.00	.00	.00	.00	12.60
Same-sex	.10	.00	.00	.00	6.70
Let others touch their genitals					
No	4.00	99.80	100.00	100.00	20.70
Other-sex	95.80	.20	.00	.00	60.70
Both-sexes	.20	.00	.00	.00	13.00
Same-sex	.00	.00	.00	.00	5.60
Oral sex					
No	21.50	100.00	100.00	100.00	39.50
Other-sex	78.20	.00	.00	.00	48.60
Both-sexes	.30	.00	.00	.00	6.30
Same-sex	.00	.00	.00	.00	5.60
Sexual intercourse					
No	35.70	100.00	100.00	100.00	53.70
Other-sex	64.30	.00	.00	.00	41.00
Both-sexes	.00	.00	.00	.00	2.50
Same-sex	.00	.00	.00	.00	2.80

No odds ratios were presented for comparing item-response probabilities across different classes given lots of zero cell sizes. The five classes were interpreted as: “high-intensity sexual behaviors, no same-sex intimacy” (Class 1); “moderate-intensity sexual behaviors, no same-sex intimacy” (Class 2); “low-intensity sexual behaviors, no same-sex intimacy” (Class 3); and “no sexual behavior” (Class 4); and the “high-intensity sexual behaviors, some same-sex intimacy” (Class 5)

For boys, the probabilities of being in the Class 1 to Class 4 at 13.5 are 4.01%, 15.88%, 34.12%, and 45.99%, respectively. For girls, the probabilities of being in the Class 1 to Class 4 at 13.5 are 3.74%, 17.19%, 34.29%, and 44.78%, respectively. There were no significant sex differences in the probabilities of being in any classes (all *p*s > .05).

For boys, the probabilities of being in the Class 1 to Class 5 at 15.5 were 22.82%, 17.62%, 30.30%, 26.85%, and 2.41%, respectively. For girls, the probabilities of being in the Class 1 to Class 5 at 15.5 are 23.94%, 19.02%, 26.20%, 22.49%, and 8.35%, respectively. Compared with boys, girls were more likely to be in the “high-intensity sexual behaviors, some same-sex intimacy” group: relative risk ratio (RRR) = 3.16, 95% confidence interval (CI) = [2.32, 4.31], *p* < .001, but less likely to be in the “low-intensity sexual behaviors, no same-sex intimacy” (RRR = 0.82, 95% CI = [0.70, 0.95], *p* < .05) and “no sexual behavior” group (RRR = 0.80, 95% CI = [0.68, 0.94], *p* < .01). There were no sex differences in the probabilities of being in the “high-intensity sexual behaviors, no same-sex intimacy” and “moderate-intensity sexual behaviors, no same-sex intimacy” groups (all *p*s > .05).

### Latent Transition Analysis of Sexual Behavior

Transition probabilities based on the estimated model are presented in Table 4. Almost 70% adolescents from “high-intensity sexual behaviors, no same-sex intimacy” at 13.5 remained in that group at 15.5 while 17.71% of them changed to be in the “high-intensity sexual behaviors, some same-sex intimacy” group at 15.5. Around 50% of those from “moderate-intensity sexual behaviors, no same-sex intimacy” at 13.5 moved to be in the “high-intensity sexual behaviors, no same-sex intimacy” at 15.5 while 12.11% of them changed to be in the “high-intensity sexual behaviors, some same-sex intimacy” group at 15.5. More than 50% of those from “low-intensity sexual behaviors, no same-sex intimacy” and “no sexual behavior” groups at 13.5 moved toward greater engagement in moderate-intensity or high-intensity sexual activities with other-sex at 15.5.

**Table 4:** Transition probabilities from 13.5 to 15.5 years old based on the estimated model

Latent class membership at 13.5	Latent class membership at 15.5				
	Class 1 (%)	Class 2 (%)	Class 3 (%)	Class 4 (%)	Class 5 (%)
Class 1	68.48	9.35	3.39	1.07	17.71
Class 2	51.62	26.96	9.12	0.19	12.11
Class 3	23.85	26.50	35.62	8.60	5.43
Class 4	6.72	12.32	29.59	49.37	2.00

The classes were interpreted as: “high-intensity sexual behaviors, no same-sex intimacy” (Class 1); “moderate-intensity sexual behaviors, no same-sex intimacy” (Class 2); “low-intensity sexual behaviors, no same-sex intimacy” (Class 3); “no sexual behavior” (Class 4); and the “high-intensity sexual behaviors, some same-sex intimacy” (Class 5)

### Consistency Between Sexual Attraction and Sexual Behavior

**Fig. 3:**
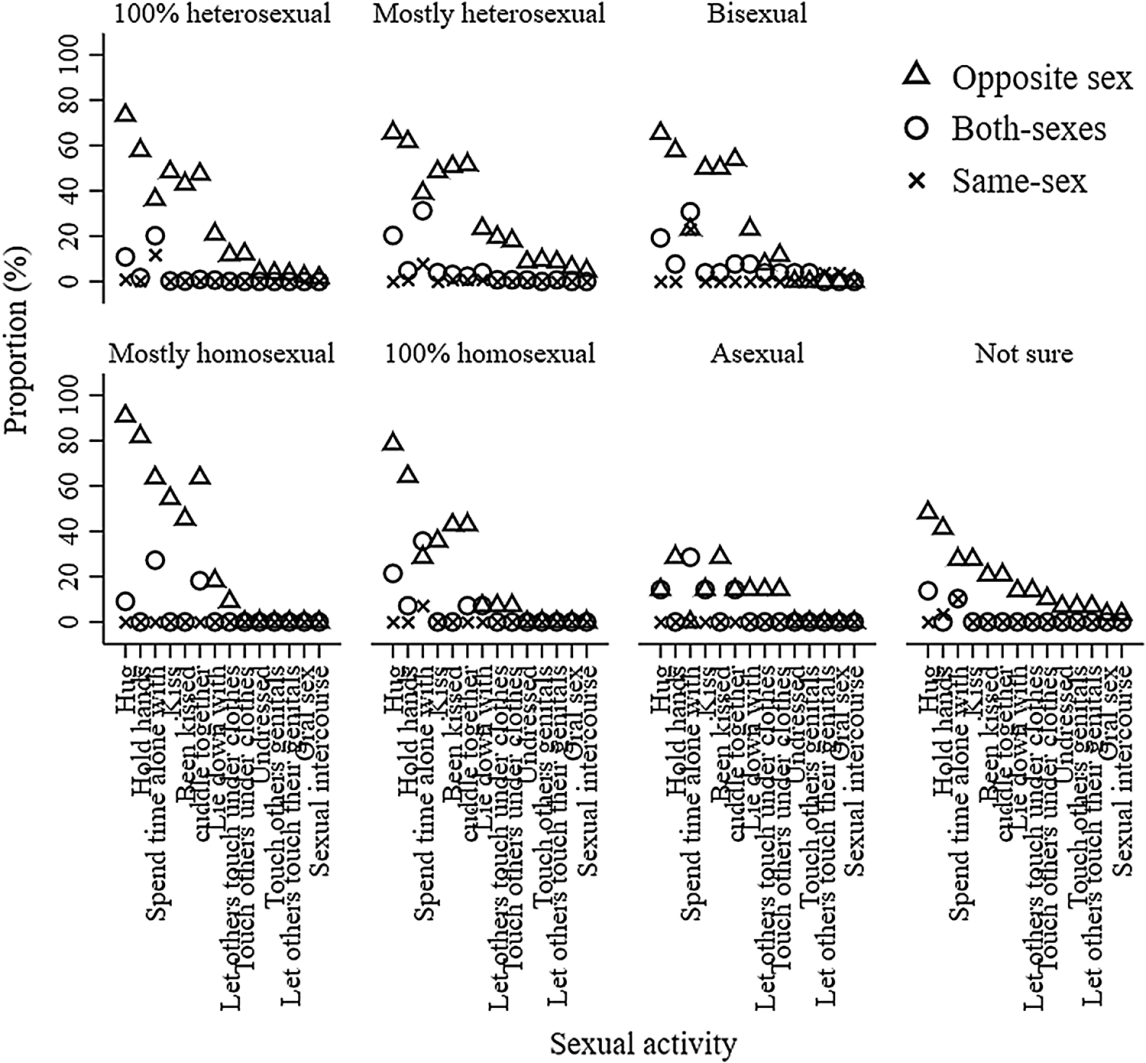
Frequency of sexual orientation by adolescent sexual behavior for boys at 13.5 years. *Note* This figure shows the probabilities of boys who engaged in sexual activity with the other-sex, both-sexes and same-sex partners in each sexual orientation groups stratified by sexual activities

**Fig. 4:**
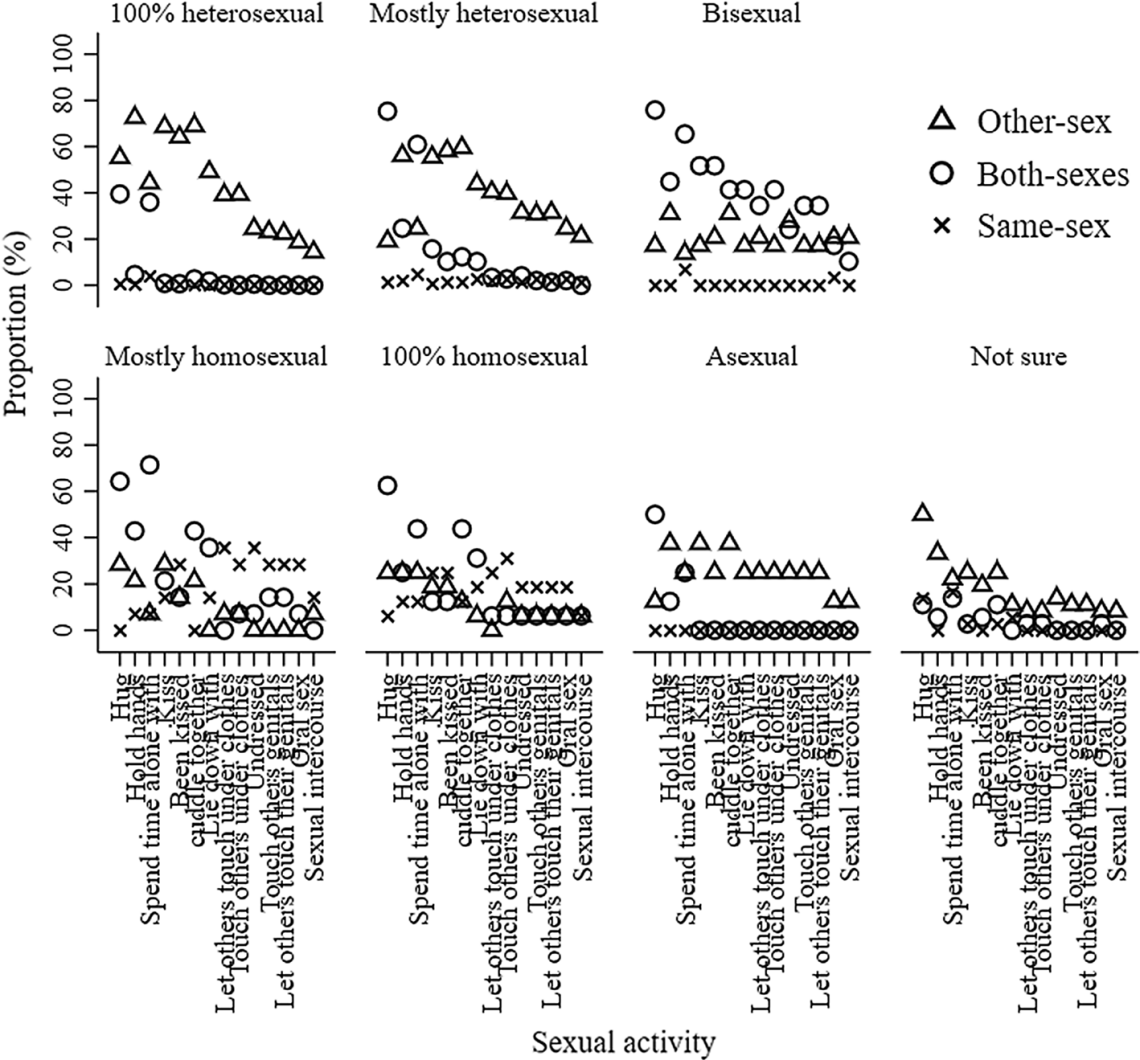
Frequency of sexual orientation by adolescent sexual behavior for boys at 15.5 years.* Note* This figure shows the probabilities of boys who engaged in sexual activity with the other-sex, both-sexes, same-sex partners in each sexual orientation groups stratified by sexual activities

Figures 3 and 4 represent the frequency of sexual orientation by adolescent sexual behaviors for boys at 13.5 and 15.5 years old, respectively. 0.00–20.18% and 0.00–39.50% of 100% heterosexual boys had engaged in sexual activities with only boys or both-sexes at 13.5 and 15.5 years old, respectively; 0.00–31.25% and 0.00–75.34% of mostly heterosexual boys had engaged in sexual activities with only boys or both-sexes at 13.5 and 15.5 years old, respectively; 0.00–65.38% and 13.79–31.03% of bisexual boys had engaged in sexual activities with only girls at 13.5 and 15.5 years old, respectively; 0.00–90.91% and 0.00– 28.57% of mostly homosexual boys had engaged in sexual activities with only girls at 13.5 and 15.5 years old, respectively; and 0.00–78.57% and 0.00–25.00% of 100% homosexual boys had engaged in sexual activities with only girls at 13.5 and 15.5 years old, respectively. 72.41% and 62.50% boys who were not sure about their sexual orientation and 71.43% and 69.44% boys who were not sexually attracted to either sex were in the “no sexual behavior” group at 13.5 and 15.5 years old, respectively. Figures 7 and 8 show the frequency of sexual orientation by latent class membership of sexual behavior in boys at 13.5 and 15.5 years old, respectively. 0.00% and 0.18% of boys from the “high-intensity sexual behaviors, no same-sex intimacy” class at 13.5 and 15.5 years old, respectively, identified themselves as 100% homosexual, and 22.41% and 27.59% of boys from the “high-intensity sexual behaviors, some same-sex intimacy” class at 15.5 years old identified themselves as 100% heterosexual and mostly heterosexual, respectively.

**Fig. 5:**
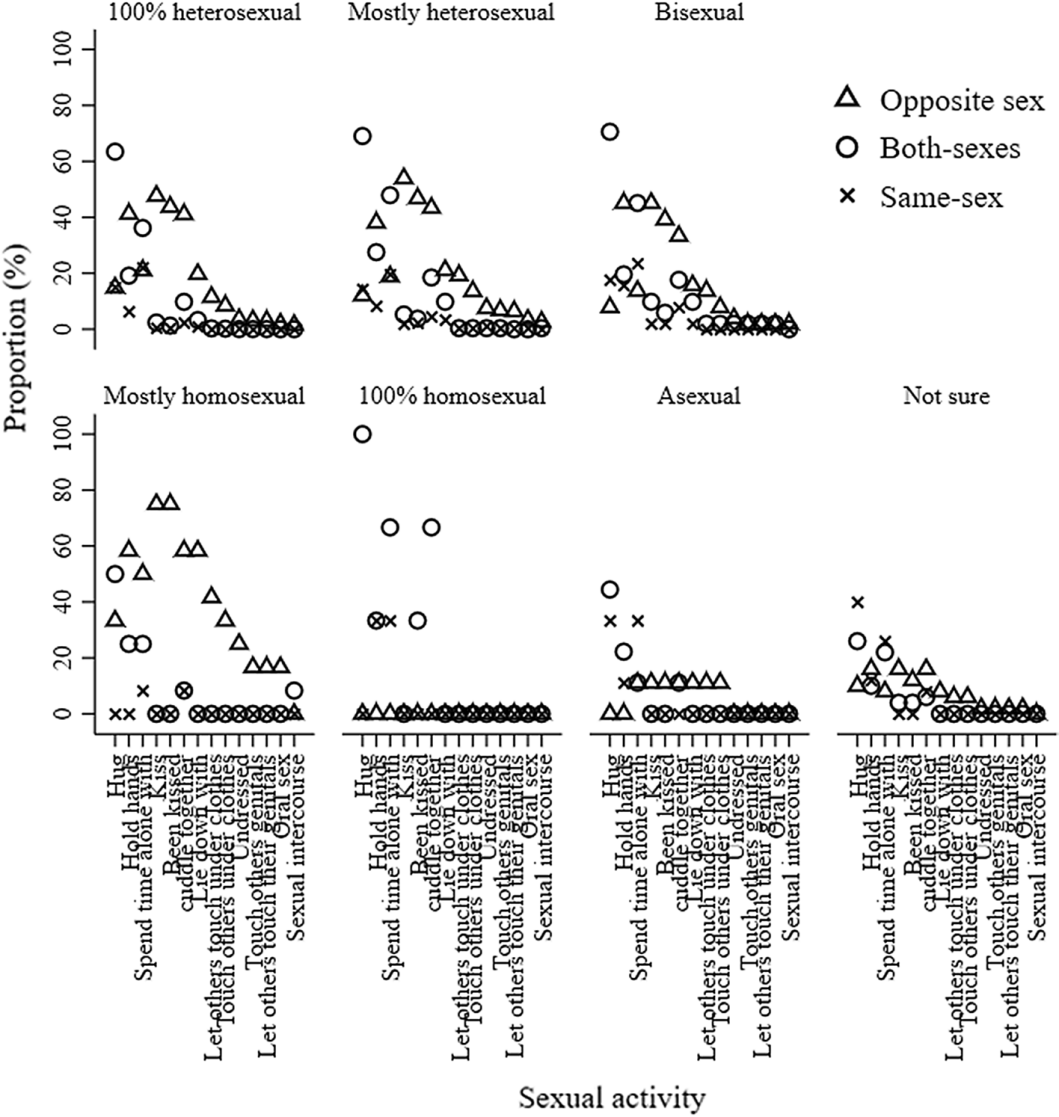
Frequency of sexual orientation by adolescent sexual behavior for girls at 13.5 years. *Note* This figure shows the probabilities of girls who engaged in sexual activity with the other-sex, both-sexes and same-sex partners in each sexual orientation groups stratified by sexual activities

**Fig. 6:**
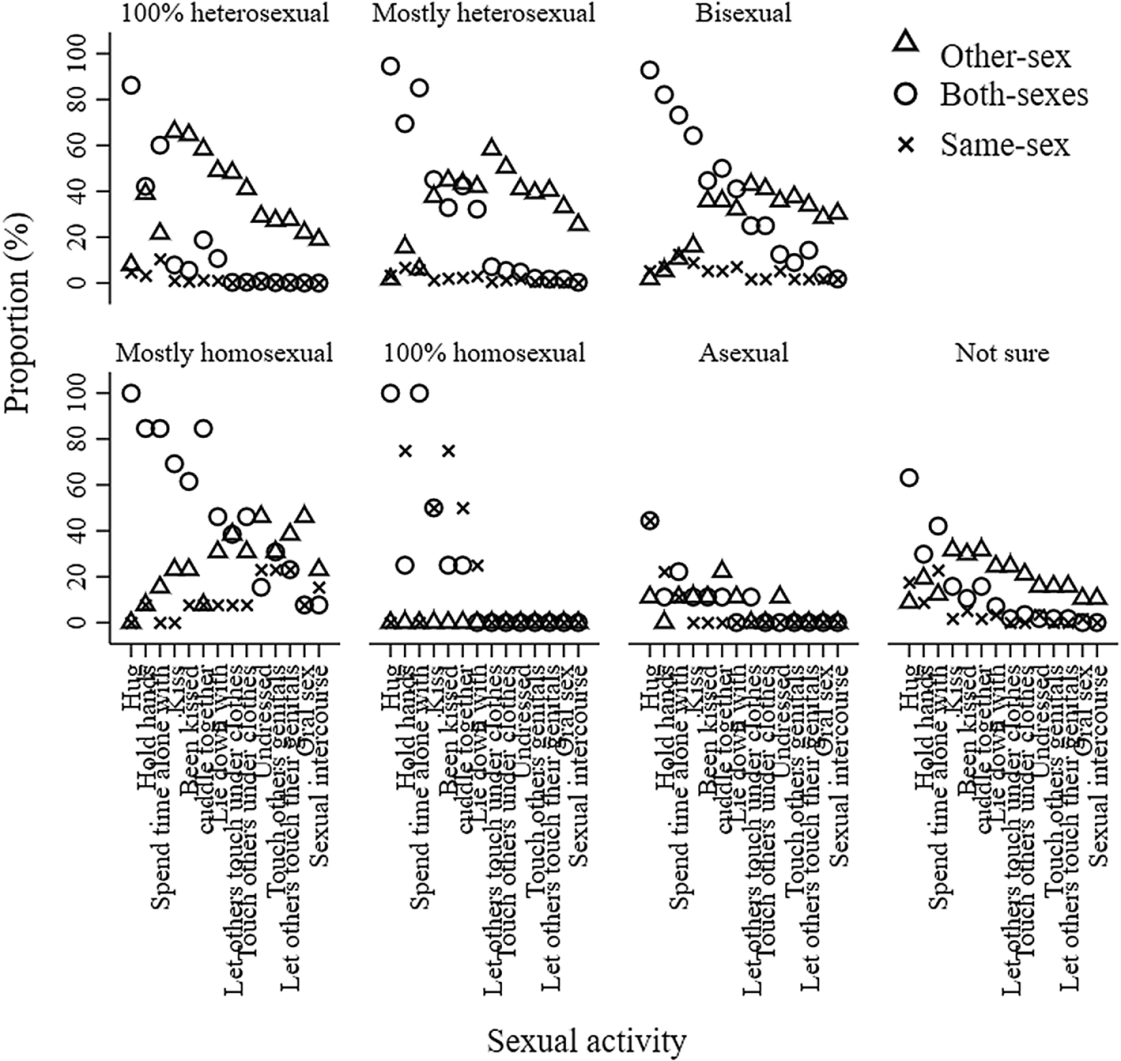
Frequency of sexual orientation by adolescent sexual behavior for girls at 15.5 years. *Note* This figure shows the probabilities of girls who engaged in sexual activity with the other-sex, both-sexes and same-sex partners in each sexual orientation groups stratified by sexual activities

**Fig. 7:**
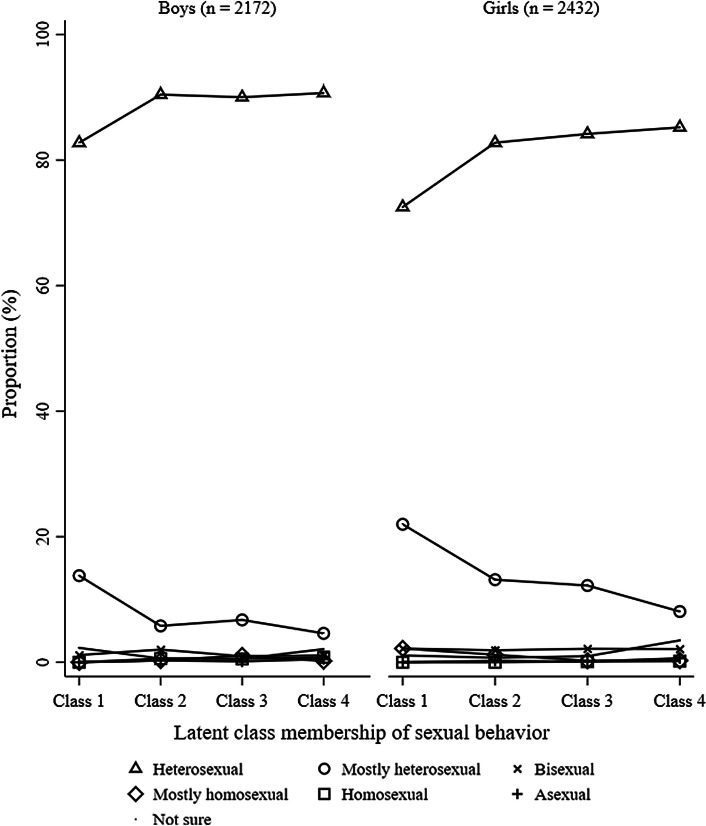
Frequency of sexual orientation by latent class membership of sexual behavior at 13.5 years. *Note* This figure shows the proportion of adolescents who identified themselves with different sexual orientation groups in each latent class membership at 13.5 years stratified by sex

**Fig. 8:**
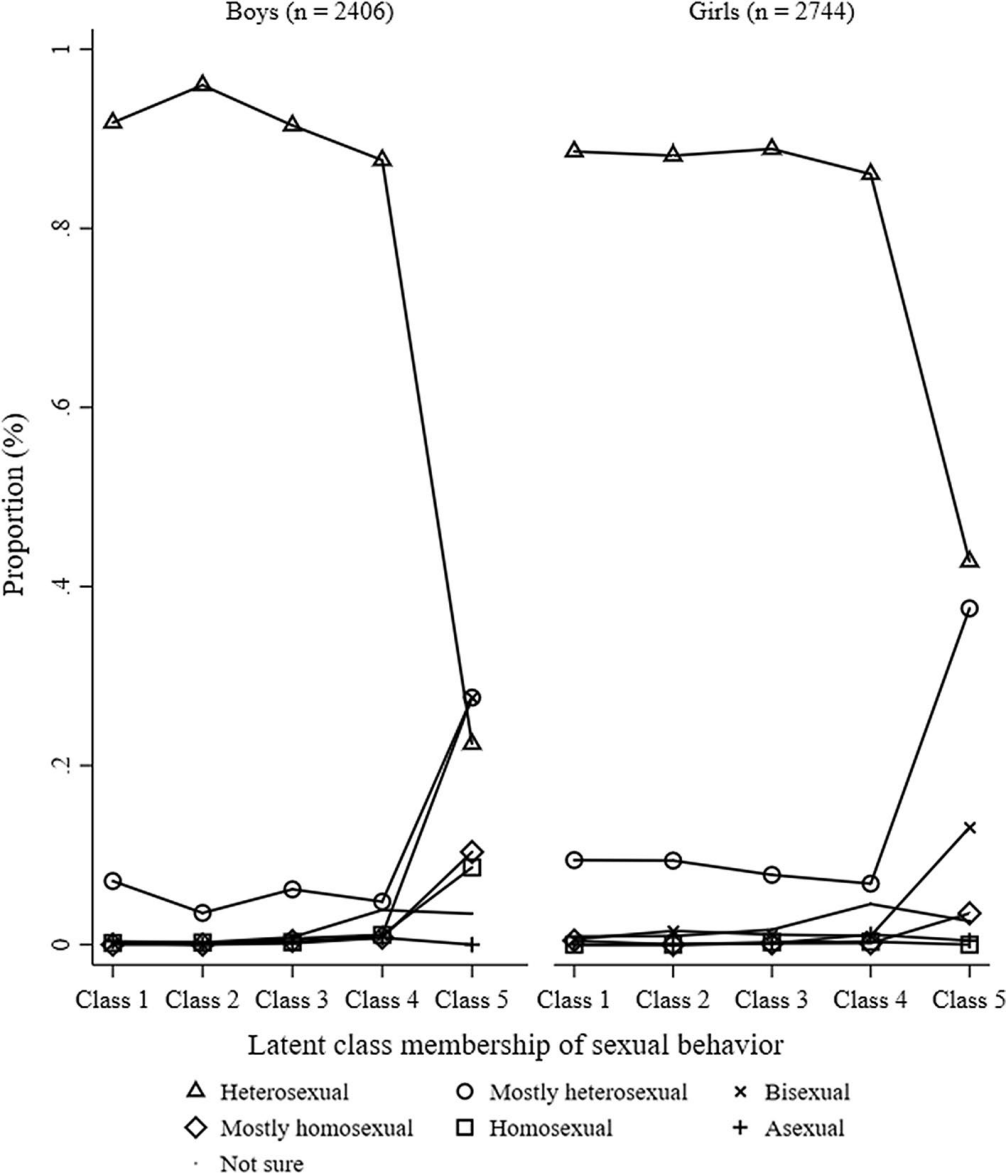
Frequency of sexual orientation by latent class membership of sexual behavior at 15.5 years. *Note* This figure shows the proportion of adolescents who identified themselves with different sexual orientation groups in each latent class membership at 15.5 years stratified by sex

Figures 5 and 6 represent the frequency of sexual orientation by adolescent sexual behaviors for girls at 13.5 and 15.5 years old, respectively. 0.00–63.47% and 0.00–86.23% of 100% heterosexual girls had engaged in sexual activities with only girls or both-sexes at 13.5 and 15.5 years old, respectively; 0.00–69.06% and 0.00–94.58% of mostly heterosexual girls had engaged in sexual activities with only girls or both-sexes at 13.5 and 15.5 years old, respectively; 1.96–45.10% and 1.79–42.86% of bisexual girls had engaged in sexual activities with only girls at 13.5 and 15.5 years old, respectively; 0.00–75.00% and 0.00–46.15% of mostly homosexual girls had engaged in sexual activities with only boys at 13.5 and 15.5 years old, respectively; and no 100% homosexual girls had engaged in sexual activities with only boys at either 13.5 or 15.5 years old. 76.00% and 49.12% girls who were not sure about their sexual orientation and 77.78% and 77.78% girls who were not sexually attracted to either sex were in the “no sexual behavior” group at 13.5 and 15.5 years old, respectively. Figures 7 and 8 also show the frequency of sexual orientation by latent class membership of sexual behavior at 13.5 and 15.5 years old, respectively. 2.20% and 0.46% of girls from the “high-intensity sexual behaviors, no same-sex intimacy” class at 13.5 and 15.5 years old, respectively, identified themselves as mostly homosexual, and 42.79% and 37.55% of girls from the “high-intensity sexual behaviors, some same-sex intimacy” class at 15.5 years old identified themselves as 100% heterosexual and mostly heterosexual.

Boys from “moderate-intensity sexual behaviors, no same-sex intimacy” (*M* = 1.13, SD = 0.49), “low-intensity sexual behaviors, no same-sex intimacy” (*M* = 1.14, SD = 0.52) and “no sexual behavior” (*M* = 1.11, SD = 0.48) groups at 13.5 years old did not differ from boys from the “high-intensity sexual behaviors, no same-sex intimacy” group at 13.5 years old (*M* = 1.16, SD = 0.40) in sexual orientation (all *p*s > .05). Compared with girls from the “high-intensity sexual behaviors, no same-sex intimacy” class at 13.5 years old (*M* = 1.33, SD = 0.64), girls from the “low-intensity sexual behaviors, no same-sex intimacy” (*M* = 1.18, SD = 0.47) and “no sexual behavior” (*M* = 1.14, SD = 0.46) groups at 13.5 years old scored as more heterosexual, Cohen’s *d* = 0.31, 95% CI = [0.09, 0.52], *p* < .05 and Cohen’s *d* = 0.40, 95% CI = [0.18, 0.61], *p* < .01, respectively. Girls from “moderate-intensity sexual behaviors, no same-sex intimacy” (*M* = 1.21, SD = 0.53) at 13.5 years old did not differ from girls from the “high-intensity sexual behaviors, no same-sex intimacy” class at 13.5 years old in sexual orientation (*p* = .089).

Compared with boys from the “high-intensity sexual behaviors, no same-sex intimacy” class (*M* = 1.08, SD = 0.32) at 15.5 years old, boys from the “high-intensity sexual behavior, some same-sex intimacy” class (*M* = 2.54, SD = 1.22) and “no sexual behavior” groups (*M* = 1.14, SD = 0.58) at 15.5 years old scored as more non-heterosexual, Cohen’s *d* = 3.05, 95% CI = [2.72, 3.37], *p* < .001 and Cohen’s *d* = 0.13, 95% CI = [0.01, 0.24], *p* < .05, respectively. Boys from “moderate-intensity sexual behaviors, no same-sex intimacy” (*M* = 1.04, SD = 0.27) and “low-intensity sexual behaviors, no same-sex intimacy” (*M* = 1.10, SD = 0.40) groups at 15.5 years old did not differ from boys from the “high-intensity sexual behaviors, no same-sex intimacy” group at 15.5 years old in sexual orientation (all *p*s > .05).

Compared with girls from the “high-intensity sexual behaviors, no same-sex intimacy” class at 15.5 years old (*M* = 1.12, SD = 0.38), girls from the “high-intensity sexual behavior, some same-sex intimacy” class at 15.5 years old (*M* = 1.77, SD = 0.82) scored as more non-heterosexual, Cohen’s *d* = 1.23, 95% CI = [1.07, 1.39], *p* < .001. Girls from “moderate-intensity sexual behaviors, no same-sex intimacy” (*M* = 1.13, SD = 0.38), “low-intensity sexual behaviors, no same-sex intimacy” (*M* = 1.12, SD = 0.41) and “no sexual behavior” classes (*M* = 1.11, SD = 0.41) at 15.5 years old did not differ from girls from the “high-intensity sexual behaviors, no same-sex intimacy” class at 15.5 years old in sexual orientation (all *p*s > .05).

## Discussion

This study using data from a birth cohort in England produced three main findings. Firstly, there were four subgroups among adolescent sexual behaviors at 13.5 years old: a “low-intensity sexual behaviors, no same-sex intimacy” group who had a high probability of kissing other-sex partners only; a “moderate-intensity sexual behaviors, no same-sex intimacy” group who had a high probability of kissing and touching with other-sex partners only; a “high-intensity sexual behaviors, no same-sex intimacy” group who had a high probability of engaging in all sexual activities exclusively with other-sex partners; and a “no sexual behavior” group who had a high probability of reporting having not engaged in any sexual activities. There was a new group found on 15.5 years old, the “high-intensity sexual behaviors, some same-sex intimacy” group which was marked by a high probability of engaging in high-intensity behaviors exclusively with the other-sex partners such as genital touching and oral sex, and low-intensity sexual activities with both-sexes partners. Secondly, around half the adolescents who have not engaged in high-intensity sexual activities at 13.5 have moved toward greater engagement in more intense sexual activities with other-sex at 15.5. Thirdly, boys and girls who were in the “high-intensity sexual behaviors, no same-sex intimacy,” “moderate-intensity sexual behaviors, no same-sex intimacy” and “low-intensity sexual behaviors, no same-sex intimacy” groups were predominantly attracted to the other-sex, whereas there was moderate consistency between low-intensity same-sex behavior and same-sex attraction for boys and low consistency for girls.

The five subgroups among adolescent sexual behaviors revealed in the current study further support the diversity of sexual behavior at middle adolescence (15.5 years measured here). The five groups identified here were very similar to the three sexual development trajectories found in a Dutch longitudinal study (Dalenberg et al., 2018). Adolescents who were in the “no sexual behavior” group may follow the non-active sexual trajectory reported by Dalenberg et al. Adolescents who were in the “low-intensity sexual behaviors, no same-sex intimacy” and “moderate-intensity sexual behaviors, no same-sex intimacy” groups may be in the early stage of a gradually sexually active trajectory, while adolescents who were in the “high-intensity sexual behaviors, no same-sex intimacy” and “high-intensity sexual behaviors, some same-sex intimacy” groups may be consistent with a fast sexually active trajectory. The latent class analysis reported here is exploratory in nature and may produce different results if sexual activities were measured at even later ages (e.g., early adulthood). Prior studies have found that the number of people who had engaged in sexual intercourse increases from adolescence to young adulthood (Shtarkshall et al., 2009). For example, about one third of participants have engaged in sexual intercourse by age 15 years, and over 90% have engaged in sexual intercourse by age 19 years in a nationally representative sample of American young adults aged 18 to 26 years (Kaestle, Halpern, Miller, & Ford, 2005). Another study using the National Longitudinal Survey of Adolescent Health data (Add Health) has found that 0.4% of boys had same-sex sexual intercourse at Wave 1 (mean age = 15.8 years), while 1.3% had same-sex intercourse at Wave 3 (mean age = 21.7 years) (Savin-Williams & Ream, 2007). Indeed, we found evidence of a new “high-intensity sexual behaviors, some same-sex intimacy” group at 15.5 compared to age 13.5 and more than 50% of those from “low-intensity sexual behaviors, no same-sex intimacy” and “no sexual behavior” groups at 13.5 moved toward greater engagement in moderate-intensity or high-intensity sexual activities with other-sex at 15.5. However, the rates of moderate to high-intensity sexual behaviors with both-sexes or same-sex were still low. Only 0.29% of adolescents (*n* = 15) had engaged in sexual intercourse with same-sex or both-sexes partners at age of 15.5 years in our sample. We expect that as adolescents become older they will transition from low-intensity sexual activities with both-sexes toward greater engagement in sexual activities with same-sex or both-sexes partners resulting in different latent growth curve profiles. Future studies using several waves of sexual behavior and attraction data at later ages could adopt latent transition analysis to further quantify the change or stability of subgroups of sexual behaviors across ages.

Over 95% boys and girls who were in the “high-intensity sexual behaviors, no same-sex intimacy,” “moderate-intensity sexual behaviors, no same-sex intimacy” and “low-intensity sexual behaviors, no same-sex intimacy” groups at both 13.5 and 15.5 identified themselves as heterosexual (100% heterosexual and mostly heterosexual), which suggests that there is strong consistency among sexual orientation components among heterosexual adolescents in our sample. Prior studies measuring sexual behavior have mainly focused on assigning sexual orientation via sexual intercourse (e.g., Eisenberg & Resnick, 2006). These studies may suffer from substantial misclassification and reductions in statistical power due to the exclusion of adolescents who only engage in low-intensity sexual activities but still self-identify as heterosexuals. Thus, future studies should consider including measurements of low-intensity sexual activities, especially for young adolescents, in order to improve the precision estimates of sexual orientation group membership.

In the contrast, there was moderate consistency between low-intensity same-sex behavior and same-sex attraction for boys and low consistency for girls. Only 46.55% of boys and 16.59% of girls who were in the “high-intensity sexual behaviors, some same-sex intimacy” group at 15.5 identified themselves as non-heterosexuals including bisexual, mostly homosexual and 100% homosexual. Prior research has found that the number of people who identified themselves as non-heterosexual increases from adolescence to adulthood (Austin et al., 2009). For example, a study using the Add Health has found that 2.5% of boys identified themselves as non-heterosexual at Wave 3, while 2.9% identified themselves as non-heterosexual at Wave 4 (Savin-Williams, Joyner, & Rieger, 2012). Thus, there is the potential for substantial misclassification and the consistency may increase from adolescent to adulthood. In addition, adolescents who were in the “high-intensity sexual behaviors, some same-sex intimacy” group may include “mostly heterosexuals.” Mostly heterosexuals were suggested to be more same-sex oriented than 100% heterosexuals but less same-sex oriented than bisexuals in terms of sexual attraction, romantic attraction and sexual behaviors (Savin-Williams & Vrangalova, 2013). Another study has found that mostly heterosexuals of both-sexes were significantly more likely to have at least one same-sex partner than did heterosexuals, but less likely to do so than did bisexuals (Saewyc et al., 2009). In addition, studies have also found that more women tend to select mostly heterosexual identities than do men (Lindley, Walsemann, & Carter, 2012; Vrangalova & Savin-Williams, 2012). Indeed, 37.55% girls and 27.59% boys from the “high-intensity sexual behaviors, some same-sex intimacy” at 15.5 identified themselves as mostly heterosexual. This may explain the relatively lower percent of girls who were in the “high-intensity sexual behaviors, some same-sex intimacy” group identified themselves as non-heterosexuals.

There are several strengths and limitations of our study. The fourteen sexual activities measured in the present study enable us to explore the diversity of adolescent sexual development and increase statistical power due to the inclusion of adolescents who only have engaged in low-intensity sexual activities. The latent class approaches allow us to use the full range of the observed sexual behavior variables, with minimal loss of data. In general, the ALSPAC cohort is well-characterized and relatively representative of the UK population permitting some generalizability. However, the present study is limited by potential misclassification due to the sexual orientation measurement at young age and the small sample sizes of boys and girls who reported having engaged in high-intensity sexual activities. The sample sizes of non-heterosexual boys and girls were small. However, given the low prevalence of non-heterosexual orientation among the population, small numbers of non-heterosexuals in cohort and population studies are to be expected. Despite the small number of non-heterosexuals, the power to detect meaningful associations was acceptable due to the large overall size of the sample. Critically, the current study measured sexual orientation when adolescents were 15.5 years of age. This age appears appropriate to begin measuring sexual orientation based on the psychometric properties of the 5-point measure used (Ott et al., 2011). However, the adolescents may change their sexual orientation reports if we reassess the cohort at later ages, which may produce somewhat different results. We examined class memberships at early and middle stage of adolescence. Future research should aim to use methods like latent transition analysis to examine transitions in class membership from early to late stage of adolescence or even early stage of adulthood (Lanza & Collins, 2008). Adolescents may also misreport their sexual orientation adding further to the problem of misclassification (Savin-Williams & Joyner, 2014). Future studies may also wish to explore associations between sexual behavior latent classes and sexual orientation measured using more objective methods such as genital arousal or pupil dilation. Finally, we were unable to test for any differences associated with ethnicity, migration status or the urban versus rural geographical divide. There may be important ethnic, geodemographic and cultural differences in sexual norms which need to be considered in future research.

In sum, the results of the present study offer support to the notion that there is a diversity of sexual activity patterns among adolescents. In addition, our results suggest there is moderate consistency between low-intensity same-sex behavior and same-sex attraction for boys and low consistency for girls. Finally, the results suggest that it is important to include low-intensity sexual behaviors to increase statistical power and reduce misclassification when assigning adolescents to sexual orientation categories using sexual behaviors.

## Electronic supplementary material

Below is the link to the electronic supplementary material.

Supplementary material 1 (PDF 243 kb)
