# Supplementary material for: Adolescent Sexual Behavior Patterns in a British Birth Cohort: A Latent Class Analysis
Source: Arch Sex Behav. 2020 Jan 6;50(1):161–80. doi: 10.1007/s10508-019-01578-w (PMC7878235; doi:10.1007/s10508-019-01578-w)
Supplement: Supplementary file 1 — Supplementary material 1 (PDF 243 kb) [file 10508_2019_1578_MOESM1_ESM.pdf]

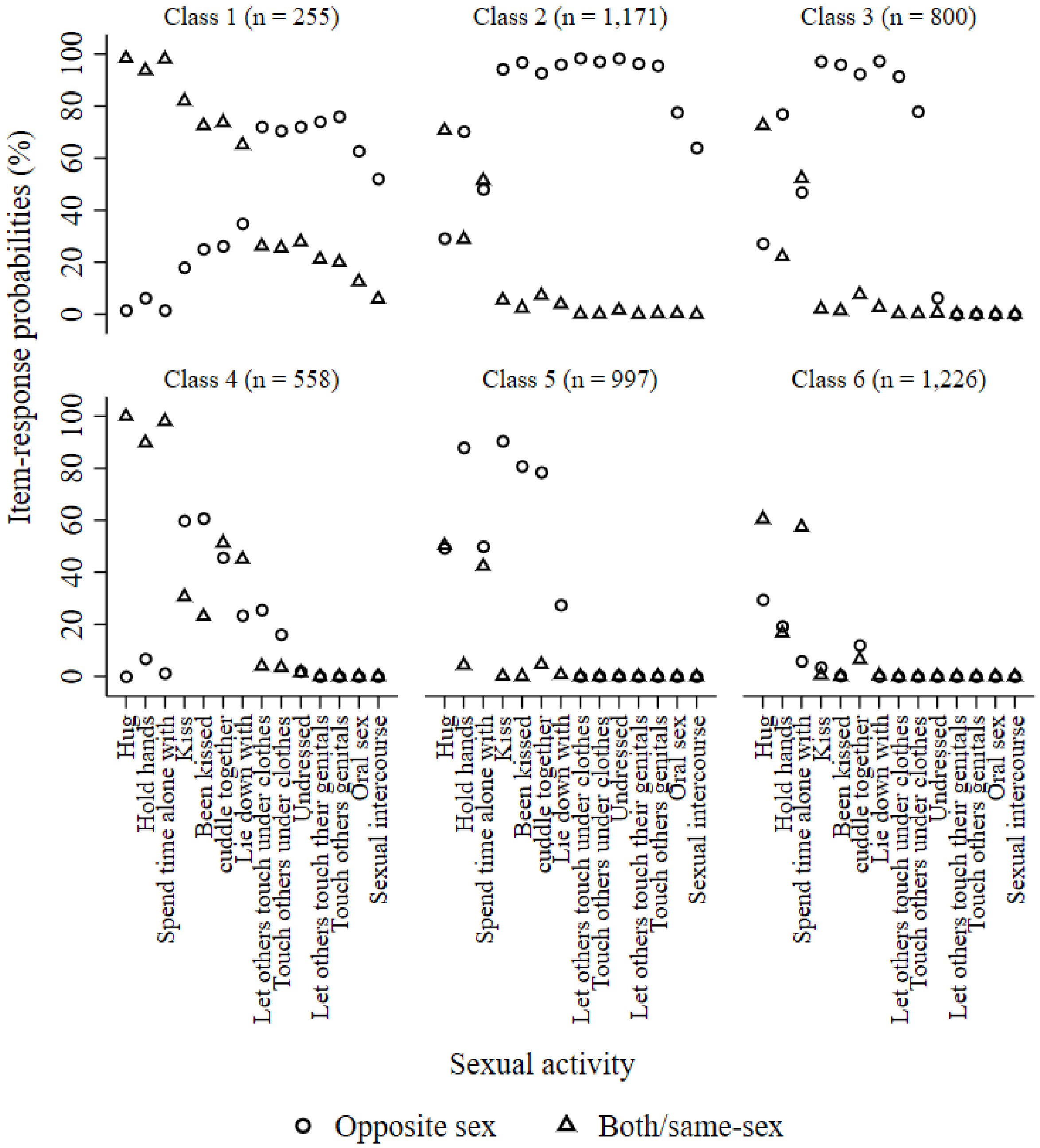

Supplemental Figure 1. Item-response probabilities for six class models of adolescent sexual behavior

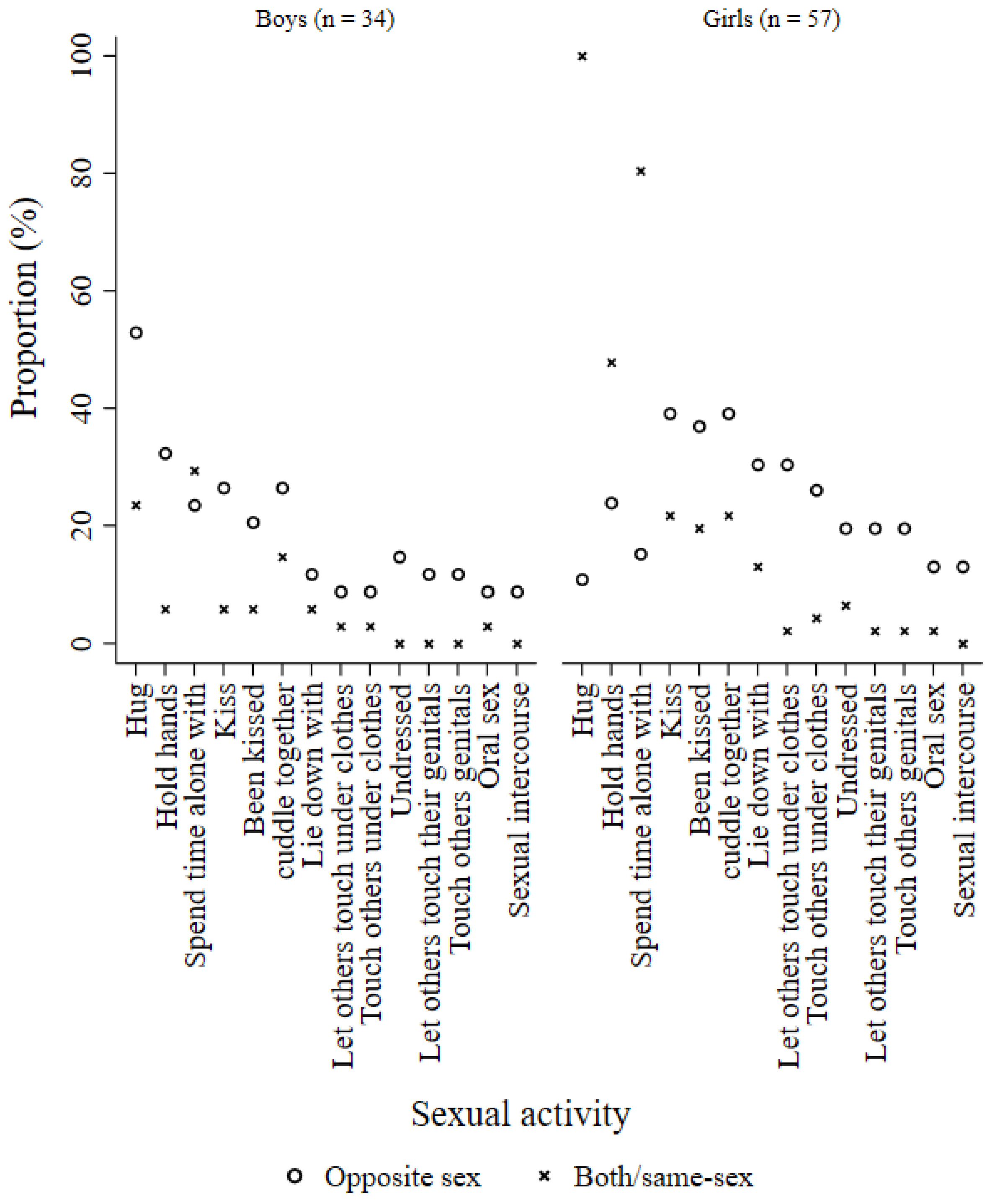

Supplemental Figure 2. Adolescents sexual behavior for “not sure” stratified by sex
